# Clinical Comparison of Beam-Specific Planning Target Volume Single-Field Uniform Dose and Robust Intensity-Modulated Proton Therapy in Ultra-Hypofractionation for Prostate Cancer

**DOI:** 10.1016/j.ijpt.2026.101951

**Published:** 2026-07-15

**Authors:** Hiroshi Tamura, Takaaki Yoshimura, Keiji Nakazato, Hiroto Yoshimoto, Seishin Takao, Taeko Matsuura, Takashi Mori, Kentaro Nishioka, Keiji Kobashi, Takayuki Hashimoto, Hidefumi Aoyama

**Affiliations:** 1 Department of Radiological Technology, Hokkaido University Hospital, Sapporo, Japan; 2 Department of Medical Physics, Hokkaido University Hospital, Sapporo, Japan; 3 Department of Health Sciences and Technology, Faculty of Health Sciences, Hokkaido University, Sapporo, Japan; 4 Graduate School of Biomedical Science and Engineering, Hokkaido University, Sapporo, Japan; 5 Faculty of Engineering, Hokkaido University, Sapporo, Japan; 6 Department of Radiation Oncology, Hokkaido University Hospital, Sapporo, Japan; 7 Global Center for Biomedical Science and Engineering, Faculty of Medicine, Hokkaido University, Sapporo, Japan; 8 Department of Radiation Oncology, Faculty of Medicine, Hokkaido University, Sapporo, Japan

**Keywords:** Prostate cancer, Robustness, Ultra-hypofractionated proton therapy, Single-field uniform dose, Intensity-modulated proton therapy

## Abstract

**Purpose:**

This study aimed to compare the robustness of beam-specific planning target volume (bs-PTV)-based single-field uniform dose (SFUD) and robustly optimized intensity-modulated proton therapy (IMPT) plans against setup, range, and anatomical uncertainties in ultra-hypofractionated (UHF) proton therapy for localized prostate cancer.

**Methods:**

SFUD and IMPT plans were generated and compared using imaging datasets from ten consecutive patients who underwent proton therapy for prostate cancer at our institution. SFUD plans using a bs-PTV and IMPT plans using robust optimization were generated with 36.25 Gy equivalent (GyE) to the clinical target volume (CTV) in 5 fractions. To evaluate robustness against setup and range uncertainties, 20 error scenarios were applied, including combined ±3 mm isocenter shifts and ±3.5% Hounsfield Unit (HU) variations. Additionally, the SFUD and IMPT plans were recalculated using virtual CT (vCT) derived from weekly cone-beam CT (wCBCT) to estimate accumulated doses under anatomical changes. The worst-case and accumulated doses to the CTV, rectum, and bladder were compared.

**Results:**

Both SFUD and IMPT plans demonstrated stable target coverage under setup, range, and anatomical uncertainties. Variations in CTV D99 remained within commonly accepted robustness criteria for all patients. Although a statistically significant difference in worst-case CTV D99 was observed between SFUD and IMPT plans, the magnitude of this difference was small. Dose variations under uncertainties were smaller for selected organs at risk (OAR) dose metrics in IMPT. Across the nominal, worst-case, and accumulated dose evaluations, IMPT plans achieved lower low- and intermediate-dose metrics for the rectum and bladder.

**Conclusion:**

Both bs-PTV-based SFUD and robustly optimized IMPT plans maintained adequate target coverage under uncertainties in UHF proton therapy for prostate cancer. The lower OAR doses and slightly improved OAR robustness observed with IMPT likely reflect the combined effects of differences in planning techniques and uncertainty management strategies, suggesting a modest dosimetric benefit.

## Introduction

In radiotherapy for prostate cancer, conventional fractionated (CF) schedules of 2 Gy per fraction to 74 to 78 Gy have traditionally been used.^1^, ^2^ Moderate-hypofractionated (MHF) schedules, such as 60 Gy in 20 fractions, have been shown to be non-inferior to conventional schedules,^3^, ^4^ and ultra-hypofractionated (UHF) schedules with higher dose per fraction (>5 Gy) have gained considerable interest in the last decade.^5^, ^6^, ^7^, ^8^, ^9^ The PACE-B trial, which used photon beams, reported a significantly higher incidence of late toxicity in the UHF schedule than the CF schedule.^7^ Late toxicity is a critical concern for patients with prostate cancer who are expected to live long after treatment, as it can significantly reduce quality of life. Doses to organs at risk (OAR) should be minimized to reduce adverse events in UHF schedules.

Proton therapy can reduce the dose to OAR while maintaining target coverage compared with photon therapy.^10^ Recently, spot-scanning proton therapy has been increasingly adopted as a means of reducing dose to the OAR,^11^ with 2 commonly applied planning techniques: single-field uniform dose (SFUD) and intensity-modulated proton therapy (IMPT).^12^, ^13^ SFUD delivers a uniform dose to the target from each individual field^12^ and is generally considered more robust to uncertainties. In contrast, IMPT simultaneously optimizes spots from all fields, providing greater flexibility in dose shaping and the potential for improved OAR sparing.^13^ However, due to its highly modulated dose distribution, IMPT has been shown to be more sensitive to setup, range, and anatomical uncertainties.^14^, ^15^, ^16^ These effects may be amplified under UHF schedules with higher per-fraction doses.

In modern clinical practice, robust optimization has been increasingly applied to both SFUD and IMPT to account for uncertainties. However, SFUD has traditionally relied on a beam-specific planning target volume (bs-PTV) that incorporates setup and range margins added to the clinical target volume (CTV),^17^ whereas in IMPT, robust optimization is commonly employed to maintain target coverage.^18^, ^19^, ^20^ Kirk et al compared the robustness of SFUD and IMPT plans against anatomical changes with a CF schedule for 10 patients with prostate cancer and found that SFUD was more robust to anatomical changes.^21^ However, their IMPT plans were generated based on the bs-PTV. Robustly optimized IMPT has since been shown to provide greater robustness than the bs-PTV approach.^22^, ^23^ We therefore hypothesized that robustly optimized IMPT would achieve robustness comparable to traditional bs-PTV-based SFUD under anatomical changes during the treatment period.

Cone-beam CT (CBCT) images acquired for patient setup can also be used to evaluate anatomical changes during the treatment period, including variations in OAR shape and tumor size. CBCT images have been widely used to characterize daily anatomical changes and to support dose estimation within the regions covered by the CBCT field of view (FOV).^24^, ^25^, ^26^, ^27^

At our institution, traditional SFUD utilizes the bs-PTV approach, whereas IMPT incorporates robust optimization. Therefore, evaluating these specific combinations is essential to comprehensively assess their performance and practical clinical utility. This study aimed to compare the robustness of bs-PTV-based SFUD and robustly optimized IMPT plans in prostate cancer under uncertainty conditions using both perturbed dose simulation and CBCT-based accumulated dose estimation.

## Methods

### Patient data

This study utilized imaging datasets from ten consecutive patients who had previously undergone proton therapy for prostate cancer at our institution. The patient characteristics were as follows: the median age was 73 years (range, 66-85 years), and 2 patients (20%) received androgen deprivation therapy. According to the National Comprehensive Cancer Network risk classification,^28^ 2 patients (20%) were classified as high-risk, 7 (70%) as unfavorable intermediate-risk, and one (10%) as favorable intermediate-risk. Each dataset consisted of planning CT (pCT) images acquired using a CT scanner (SOMATOM Confidence RT Pro; Siemens Healthineers, Forchheim, Germany) and between 5 and 8 weekly CBCT (wCBCT) images acquired using a CBCT system integrated with a proton delivery system (PROBEAT-RT; Hitachi, Tokyo, Japan). The first 5 wCBCT scans were used for the present analysis. These images were acquired weekly before treatment to monitor rectal and bladder filling during the MHF schedule delivered in 21 fractions.　This study was approved by our institutional review board.

All patients underwent both pCT and magnetic resonance imaging (MRI) for treatment planning. One week before imaging, 3 1.5-mm diameter gold markers (iGold; MEDIKIT, Tokyo, Japan) were inserted into the prostate, and a 10-mL hydrogel spacer (SpaceOAR; Augmenix Inc, Waltham, MA) was injected posterior to the prostate.^29^ Patients were immobilized in the supine position using a vacuum cushion (Vac-Lok; CIVCO Medical Solutions, Kalona, IA, USA), with their arms positioned on the chest during pCT acquisition. The pCT scan protocol was as follows: 120 kVp, 450 mAs, 1 mm slice thickness, 500 × 500 mm FOV, and a transverse pixel size of 0.98 × 0.98 mm. Patients were instructed to drink 200 mL of water and to refrain from urination and defecation for 1 hour before pCT acquisition and beam delivery. In all cases, CBCT images were acquired weekly prior to beam delivery to monitor bladder filling and rectal gas. The CBCT scan protocol used half-scan mode at 125 kVp and 640 mAs, with 2 mm slice thickness, 200 × 200 mm FOV, and 0.42 × 0.42 mm transverse pixel size.

### Nominal treatment planning

Experienced radiation oncologists delineated the prostate, seminal vesicles (SV), rectum, bladder, and femoral head on the pCT images. Urethral delineation was performed on the pCT images based on MRI-to-pCT image registration for 9 patients in whom the prostatic urethra was visible on MRI.^30^ Artifacts generated by the gold markers were overridden with surrounding soft tissue densities for dose calculation. The CTV was defined as the prostate and the proximal 1 cm of the SV, assuming intermediate-risk patients for the purpose of this study.

A total of 20 plans (10 SFUD and 10 IMPT plans) were generated using the VQA treatment planning system (Hitachi Ltd, Hitachi, Japan) employing a pencil beam algorithm and a prescribed dose of 36.25 Gy equivalent (GyE) in 5 fractions. The dose was calculated using a relative biological effectiveness (RBE) of 1.1 with a dose grid of 2 mm. The spot size was determined by the machine commissioning data for each energy layer (70-220 MeV). The spot spacing at the isocenter was 5.3 to 6.1 mm, with no substantial difference observed between the SFUD and IMPT plans. All plans consisted of 4 fields with gantry angles of 75°, 100°, 260°, and 285°, equally weighted to minimize the dosimetric effects of the implanted gold markers.^31^ All plans were optimized to deliver 36.25 GyE to the CTV (evaluation criterion: CTV D99% ≥ 36.25 GyE). Dose constraints for OAR were applied as follows: rectum: V18.1 GyE < 50%, V29 GyE < 20%, V36 GyE < 1 cc; bladder: V18.1 GyE < 40%, V37 GyE < 10 cc; femoral head: V14.5 GyE < 5%, based on the PACE-B trial.^6^, ^7^ Although the urethra was not incorporated as a dose constraint during plan generation, its dose was retrospectively assessed using the PACE-B trial criteria (V42 GyE < 50%) to ensure the clinical feasibility of the plans.^6^, ^7^

To account for setup and range uncertainties, SFUD plans were generated with bs-PTV.^17^ The bs-PTV was created by extending the lateral margin (LM), proximal margin (PM), and distal margin (DM) from the CTV. The LM was kept constant at 3 mm perpendicular to the beam direction. PM and DM were calculated as 3.5% of the water-equivalent path from the skin to the proximal and distal edges of the CTV, with an additional 1 mm margin.^32^ In contrast, IMPT plans were generated with robust optimization based on the worst-case approach.^19^ Robust optimization was applied only to the CTV using setup and range uncertainties of 3 mm and 3.5%, respectively, to maintain target coverage under uncertainty scenarios.

### Robustness analysis of nominal plans

#### Robustness against setup and range uncertainties

During the evaluation of the robustness of nominal SFUD and IMPT plans against setup and range uncertainties, perturbed dose distributions were generated under 20 error scenarios. These consisted of 18 error scenarios combining isocenter shifts of ±3 mm along the left-right (L-R), superior-inferior (S-I), and anterior-posterior (A-P) axes with Hounsfield Unit (HU) variations of 0%, +3.5%, and −3.5% on the pCT images and 2 scenarios of isolated HU variations (±3.5% with no isocenter shift). For each of the 10 patients, all 20 error scenarios were evaluated for both the SFUD and IMPT plans. Subsequently, the worst-case value of each dose metric (Metric_worst_) among these 20 scenarios was identified for each patient and used for robustness analysis.

#### Robustness against anatomical changes

To evaluate the robustness of the SFUD and IMPT plans against anatomical changes, wCBCT images were used. In all wCBCT images, the CTV, rectum, and bladder were completely included within the FOV. However, parts of the femoral head and the external body contour were partially outside the FOV. Therefore, virtual CTs (vCTs) were generated from the pCT-wCBCT image pairs using MIM (version 7.4.2; MIM Software Inc, Cleveland, OH, USA). The CBCT-based vCT generation and dose recalculation were performed as follows:

1. Regions outside the wCBCT images were replaced with the corresponding pCT data after performing rigid registration along 6 axes between the pCT and wCBCT. Examples of wCBCT, pCT, and the replaced wCBCT are shown in Figure 1.

**Figure 1:**
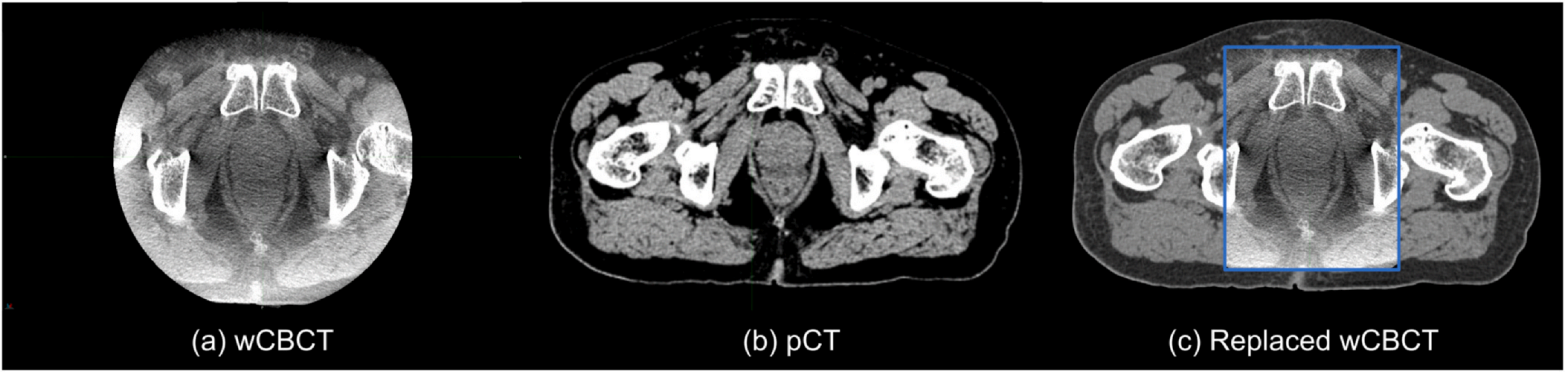
Representative images of the wCBCT (a), pCT (b), and replaced CBCT (c). In panel (c), the region inside the blue line corresponds to the wCBCT, while the region outside the blue line corresponds to the pCT.
2. The pCT was deformably registered to each of the 5 replaced wCBCTs for each patient, creating vCTs that reflected the daily anatomical features observed in the wCBCT images.^24^, ^25^, ^26^, ^27^ Contours of the pCT were propagated to the vCTs using deformable image registration (DIR) and, when necessary, manually adjusted by experienced radiation oncologists. DIR accuracy was assessed through visual inspection of anatomical landmarks and contour consistency in all cases.
3. To estimate the fractionated delivered dose, the nominal SFUD and IMPT plans were recalculated on each vCT. Positional alignment was assumed using a gold fiducial marker, and forward calculations were performed on each vCT using an in-house dose calculation program based on the same algorithm as VQA.^33^ To validate the consistency between this program and VQA, a 3D gamma analysis was performed to compare the dose distributions calculated by both systems for 3 cases (including both SFUD and IMPT plans). A gamma pass rate of 100% was achieved across all cases under the criteria of 1% and 1 mm, demonstrating excellent agreement between the 2 systems. It should be noted that this result does not necessarily guarantee dose-calculation accuracy.
4. Each of the 5 fractionated dose distributions was deformed to the pCT, and the accumulated delivered dose over the treatment period was calculated by summing them on the same pCT using MIM.^34^, ^35^ Consequently, an accumulated dose metric (Metric_accum_) was obtained for each patient for the SFUD and IMPT plans.

### Comparison between bs-PTV-based SFUD and robustly optimized IMPT plans

For both the SFUD and IMPT plans, the nominal dose metric (Metric_nominal_) was used as the reference. Plan robustness against uncertainties was evaluated by calculating the differences between the worst-case or accumulated scenarios and the nominal plan for each evaluated dose metric.

For each metric, the differences were defined as:

(1) $${∆}_{\mathrm{worst}}={\mathrm{Metric}}_{\mathrm{worst}}-{\mathrm{Metric}}_{\mathrm{nominal}}$$

(2) $${∆}_{\mathrm{accum}}={\mathrm{Metric}}_{\mathrm{accum}}-{\mathrm{Metric}}_{\mathrm{nominal}}$$

where *Metric* represents CTV D99, homogeneity index (HI), conformity index (CI), or the respective OAR dose–volume parameters. Target coverage was evaluated using the HI, CI, and CTV D99 (GyE), while OAR sparing was assessed using rectum V18.1 (%), V29 (%), and V36 (cc) and bladder V18.1 (%) and V37 (cc). Femoral head V14.5 (%) was excluded from the accumulated dose analysis because it was partially outside the wCBCT FOV. Similarly, urethra V42 (%) was also excluded from the accumulated dose analysis, as the limited soft-tissue contrast of the CBCT prevented reliable dose calculation.

The HI and CI were calculated as follows^36^, ^37^:

(3) $$\mathrm{HI}=\frac{D2-D98}{D50}$$

(4) $$\mathrm{CI}=\frac{\mathrm{CTV}\cap V36.25}{\mathrm{CTV}}\times \frac{\mathrm{CTV}\cap V36.25}{V36.25}$$

where D2, D50, and D98 represent the minimum doses to 2%, 50%, and 98% of the CTV, respectively. V36.25 represents the volume receiving at least 36.25 GyE.

Paired differences in dose metrics (calculated as IMPT minus SFUD) were also evaluated for the nominal, worst-case, and accumulated doses.

### Statistics

JMP PRO (version 17.0; SAS Institute, Cary, NC, USA) was used for statistical analysis. The Wilcoxon signed-rank test was used for paired comparisons of nominal, Δ_worst_, and Δ_accum_ dose metrics between the SFUD and IMPT plans. A *P* value of less than 0.05 was considered statistically significant.

## Results

### Anatomical changes

Table 1 shows the median and range (minimum-maximum) of volume changes in the CTV, rectum, and bladder between the pCT and vCT over 5 fractions for each patient. Across patients, the median volume changes between the pCT and vCT were −0.03% (range −7.04% to 4.46%) for the CTV, 8.93% (−16.05% to 85.16%) for the rectum, and 0.27% (−40.27% to 37.27%) for the bladder.

**Table 1:** Median and range (minimum-maximum) of volume changes in the CTV, rectum, and bladder between pCT and vCT for each patient.

Patient	CTV [%]			Rectum [%]			Bladder [%]		
	Median	Min	Max	Median	Min	Max	Median	Min	Max
1	−0.41	−3.32	0	30.70	−1.90	85.16	28.01	13.43	37.27
2	0.46	−0.14	0.90	36.38	6.05	47.80	1.13	−13.97	20.00
3	−0.21	−0.50	1.16	−0.74	−6.02	6.88	16.88	0.78	30.43
4	−0.28	−1.55	−0.06	10.60	3.31	35.87	10.59	6.55	21.57
5	−4.76	−5.74	−1.63	25.83	14.91	30.13	−0.24	−8.42	17.04
6	−0.25	−2.15	0.17	13.79	9.91	24.75	−16.50	−21.13	−9.54
7	1.90	−7.04	4.19	3.43	−1.14	9.32	−2.05	−9.35	15.18
8	0.77	−0.68	4.46	−1.62	−3.48	40.15	−15.05	−24.98	9.88
9	0.34	−2.94	1.05	−7.03	−16.05	23.69	−4.85	−15.06	6.64
10	1.28	1.09	3.51	5.93	3.11	9.34	−27.94	−40.27	−1.87

*Abbreviations:* CTV, clinical target volume; pCT, planning CT; vCT, virtual CT.

Supplementary Table S1 presents the median and range of the inter-fractional positional displacements of the gold marker relative to the pelvic bony anatomy in the LR, SI, and AP directions across 5 CBCT datasets for each patient.

### Evaluation of nominal plan

Table 2 summarizes the mean and standard deviation (SD) of each dose metric for the nominal bs-PTV-based SFUD and robustly optimized IMPT plans in 10 patients. Target coverage was comparable between the 2 planning paradigms, with no statistically significant differences observed in CTV D99 (GyE) and HI. Modest but statistically significant improvements in OAR sparing were observed in the IMPT plans for selected low- and intermediate-dose metrics of the rectum and bladder, including rectum V18.1 (%), rectum V29 (%), and bladder V18.1 (%). No statistically significant differences were observed for rectum V36 (cc), bladder V37 (cc), or femoral head V14.5 (%).

**Table 2:** Comparison of dose metrics (mean ± SD) between nominal bs-PTV-based SFUD and robustly optimized IMPT plans.

Dose metrics	Dose objectives and constraints	SFUD	IMPT	*P* value
CTV D99 [GyE]	>36.25	36.50 ± 0.08	36.49 ± 0.06	.9004
HI	n/a	0.02 ± 0.02	0.01 ± 0.00	.5000
CI	n/a	0.67 ± 0.05	0.69 ± 0.05	**.0313**
Rectum V18.1 [%]	<50	18.16 ± 6.53	12.78 ± 5.28	**.0020**
Rectum V29 [%]	<20	5.42 ± 5.27	3.57 ± 3.26	**.0020**
Rectum V36 [cc]	<1	0.33 ± 0.78	0.09 ± 0.15	.1563
Bladder V18.1 [%]	<40	22.54 ± 10.59	20.22 ± 9.65	**.0273**
Bladder V37 [cc]	<10	0.13 ± 0.38	0.01 ± 0.02	.9375
Urethra V42 [%]	<50*	0.00 ± 0.00	0.00 ± 0.00	1.0000
Femoral head V14.5 [%]	<5	2.81 ± 3.10	2.37 ± 2.44	1.0000

*Abbreviations:* CI, conformity index; CTV, clinical target volume; HI, homogeneity index; IMPT, intensity-modulated proton therapy; n/a, not applicable; SFUD, single-field uniform dose.

Bold text indicates statistical significance at *P* < .05 level.

* Evaluated based on the PACE-B trial (not an optimization constraint).

Under the SFUD plan, the dose constraints were not achieved for rectum V36 in 1 patient, bladder V18.1 in 1 patient, and femoral head V14.5 in 2 patients. In contrast, under the IMPT plan, dose constraint was not achieved only for femoral head V14.5 in 1 patient. The paired differences in these nominal dose metrics between the SFUD and IMPT are shown in Supplementary Figure S1.

**Figure 2:**
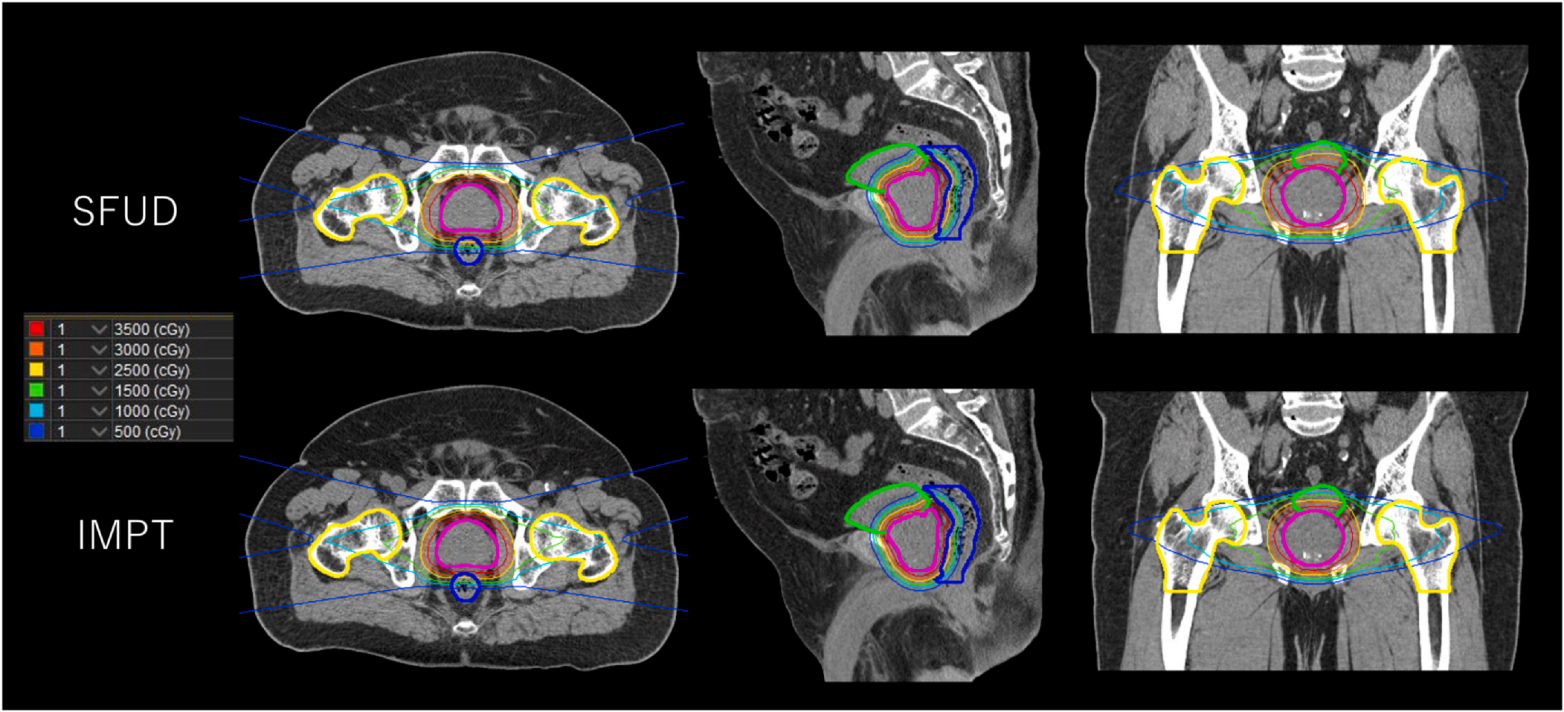
Comparison of nominal dose distributions between bs-PTV-based SFUD (upper panel) and robustly optimized IMPT (lower panel) plans from the same patient. The regions of interest (ROI) are displayed in the CTV (magenta), rectum (blue), bladder (light green), and femoral head (yellow).

Figure 2 shows nominal dose distributions for a representative patient in the SFUD and IMPT plans. The dose distributions suggested slightly improved sparing of normal tissue surrounding the prostate with the IMPT plan while maintaining comparable target coverage.

### Robustness comparison against setup and range uncertainties

Table 3 summarizes the Metric_worst_ and Δ_worst_ under setup and range uncertainties for each patient in the SFUD and IMPT plans. The Δ_worst_ of SFUD plans demonstrated significantly smaller deviations than the IMPT plans for both CTV D99 (median [range]: −1.26 [−1.75 to −0.97] GyE vs. −1.61 [−1.78 to −1.29] GyE, *P* = .0059) and HI (0.02 [0.01-0.03] vs. 0.04 [0.03-0.04], *P* = .0039). However, the worst-case dose of CTV D99 remained above 95% of the prescribed dose in all cases for both SFUD and IMPT plans. For the OAR, the deviations were significantly smaller in the IMPT plans than in the SFUD plans (rectum V18.1: 9.18 [6.25-13.72] percentage points (pp) vs. 10.27 [7.79-16.69] pp, *P* = .0137; bladder V37: 0.10 [0.02-0.60] cc vs. 1.40 [0.75-3.66] cc, *P* = .0020). Urethra V42 did not deviate from 0% in the nominal plans for all applicable patients. Although statistically significant differences were observed in some dose metrics, the magnitude of Δ_worst_ was small.

**Table 3:** Worst-case perturbed doses and Δ_worst_ under setup and range uncertainties for each patient: a comparison between the bs-PTV-based SFUD and robustly optimized IMPT plans.

Patients	Technique	CTV D99 [GyE]	HI	CI	Rectum V18.1 [% (pp)]	Rectum V29 [% (pp)]	Rectum V36 [cc]	Bladder V18.1 [% (pp)]	Bladder V37 [cc]	Urethra V42 [% (pp)]	Femoral head V14.5 [% (pp)]
1	SFUD	35.34 (−1.05)	0.05 (0.02)	0.64 (−0.03)	21.02 (7.79)	8.70 (3.67)	0.32 (0.32)	57.46 (14.91)	1.46 (1.40)	0.00 (0.00)	2.51 (0.78)
	IMPT	35.00 (−1.38)	0.04 (0.03)	0.72 (−0.01)	16.74 (6.25)	7.92 (3.83)	0.52 (0.47)	54.16 (15.82)	0.08 (0.08)	0.00 (0.00)	1.59 (0.42)
2	SFUD	35.34 (−1.07)	0.03 (0.02)	0.67 (−0.09)	30.81 (13.75)	10.47 (8.27)	0.00 (0.00)	45.10 (7.77)	0.75 (0.75)	0.00 (0.00)	9.12 (1.99)
	IMPT	35.13 (−1.29)	0.04 (0.03)	0.74 (−0.02)	23.03 (11.89)	8.04 (6.99)	0.00 (0.00)	40.70 (8.46)	0.03 (0.03)	0.00 (0.00)	9.58 (1.51)
3	SFUD	35.08 (−1.36)	0.08 (0.01)	0.56 (−0.02)	44.83 (10.62)	28.02 (9.39)	5.66 (3.15)	26.79 (7.62)	4.87 (3.66)	0.00 (0.00)	2.56 (0.76)
	IMPT	34.90 (−1.63)	0.05 (0.04)	0.68 (−0.01)	34.98 (11.69)	19.53 (9.81)	2.29 (2.13)	29.30 (8.41)	0.47 (0.40)	0.00 (0.00)	5.13 (0.98)
4	SFUD	34.76 (−1.75)	0.04 (0.03)	0.65 (−0.08)	34.66 (13.14)	13.10 (5.86)	0.67 (0.48)	33.14 (8.85)	1.73 (1.73)	0.00 (0.00)	4.81 (1.56)
	IMPT	34.75 (−1.78)	0.05 (0.04)	0.70 (−0.02)	27.13 (10.75)	11.30 (5.60)	0.68 (0.55)	31.84 (9.22)	0.42 (0.41)	0.00 (0.00)	3.37 (1.02)
5	SFUD	35.03 (−1.49)	0.04 (0.03)	0.54 (−0.06)	23.55 (10.26)	7.68 (5.84)	0.02 (0.02)	30.85 (6.60)	1.40 (1.40)	0.00 (0.00)	0.10 (0.09)
	IMPT	35.14 (−1.36)	0.04 (0.03)	0.58 (−0.01)	18.70 (8.93)	6.85 (5.42)	0.09 (0.09)	28.98 (6.86)	0.14 (0.12)	0.00 (0.00)	0.12 (0.08)
6	SFUD	35.42 (−1.19)	0.03 (0.02)	0.61 (−0.03)	29.83 (8.97)	15.16 (6.21)	1.38 (0.84)	14.72 (3.74)	2.12 (2.12)	0.00 (0.00)	0.38 (0.27)
	IMPT	34.93 (−1.65)	0.05 (0.04)	0.66 (−0.01)	26.87 (8.52)	14.30 (6.27)	1.36 (0.88)	13.60 (3.98)	0.63 (0.60)	0.00 (0.00)	2.13 (0.17)
7	SFUD	35.16 (−1.31)	0.04 (0.02)	0.64 (−0.06)	23.53 (10.28)	7.47 (6.00)	0.02 (0.02)	31.34 (8.71)	1.35 (1.35)	0.00 (0.00)	4.58 (0.37)
	IMPT	34.92 (−1.56)	0.05 (0.04)	0.69 (−0.02)	18.57 (9.42)	5.70 (5.08)	0.01 (0.01)	31.13 (9.08)	0.02 (0.02)	0.00 (0.00)	4.09 (0.32)
8	SFUD	35.29 (−1.31)	0.04 (0.02)	0.62 (−0.03)	36.46 (16.69)	10.22 (6.55)	0.33 (0.28)	17.01 (4.44)	3.09 (3.09)	0.00 (0.00)	1.40 (0.65)
	IMPT	34.86 (−1.67)	0.05 (0.04)	0.67 (−0.01)	26.63 (13.72)	7.33 (5.28)	0.20 (0.17)	16.08 (4.58)	0.02 (0.02)	0.00 (0.00)	1.30 (0.49)
9	SFUD	35.23 (−1.20)	0.04 (0.02)	0.61 (−0.04)	23.26 (8.67)	9.57 (5.97)	0.18 (0.17)	14.86 (3.98)	0.88 (0.88)	n/a	0.52 (0.32)
	IMPT	34.88 (−1.63)	0.05 (0.04)	0.65 (−0.02)	19.70 (8.10)	8.62 (5.94)	0.24 (0.24)	13.37 (4.09)	0.05 (0.04)	n/a	0.23 (0.13)
10	SFUD	35.61 (−0.97)	0.03 (0.02)	0.62 (−0.02)	22.58 (8.76)	5.47 (3.87)	0.07 (0.07)	26.77 (6.05)	1.33 (1.33)	0.00 (0.00)	9.95 (1.02)
	IMPT	34.86 (−1.59)	0.05 (0.04)	0.60 (−0.12)	12.33 (7.62)	2.77 (2.44)	0.03 (0.03)	19.06 (5.49)	0.18 (0.18)	0.00 (0.00)	2.48 (1.19)

*Abbreviations:* CI, conformity index; CTV, clinical target volume; HI, homogeneity index; IMPT, intensity-modulated proton therapy; n/a, not applicable; pp, percentage point; SFUD, single-field uniform dose.

*Notes:* Data are presented as absolute worst-case perturbed dose metrics together with the corresponding differences (Δ_worst_). For dose metrics evaluated as percentages, absolute values are expressed as %, and Δ_worst_ values are expressed in percentage points (pp).

Regarding Metric_worst_, the dose constraint of rectum V29 was not achieved in 1 patient with the SFUD plan, whereas it was achieved in all patients with the IMPT plan. Additionally, the femoral head V14.5 dose constraint was not achieved in 2 patients with the SFUD plan and 1 patient with the IMPT plan. The paired differences between SFUD and IMPT for these Metric_worst_ are presented in Supplementary Figure S1. Among the evaluated metrics, the largest difference was observed in rectum V18.1, with a median difference of −6.25 pp.

### Robustness comparison against anatomical changes

Table 4 summarizes the Metric_accum_ and Δ_accum_ under anatomical changes, evaluated using wCBCT images, for each patient in the SFUD and IMPT plans. For the CTV, deviations in dose metrics, including CTV D99, HI, and CI, were small in both SFUD and IMPT plans, with no statistically significant differences. For the OAR, a statistically significant difference in Δ_accum_ between the SFUD and IMPT plans was observed only for rectum V18.1 (−2.62 [−7.42 to 0.59] pp vs. −2.03 [−6.98 to 1.34] pp, *P* = .0020), for which the absolute deviation was smaller in the IMPT plans. For all other rectum and bladder dose metrics, including rectum V29 (%), rectum V36 (cc), bladder V18.1 (%), and bladder V37 (cc), no statistically significant differences in Δ_accum_ were observed between the SFUD and IMPT plans.

**Table 4:** Accumulated doses and Δ_accum_ under anatomical changes for each patient: a comparison between the bs-PTV-based SFUD and robustly optimized IMPT plans.

Patients	Optimization	CTV D99 [GyE]	HI	CI	Rectum V18.1 [% (pp)]	Rectum V29 [% (pp)]	Rectum V36 [cc]	Bladder V18.1 [% (pp)]	Bladder V37 [cc]
1	SFUD	35.76 (−0.63)	0.04 (0.01)	0.65 (−0.02)	10.96 (−2.27)	3.65 (−1.38)	0.00 (0.00)	32.90 (−9.65)	0.00 (−0.06)
	IMPT	35.48 (−0.90)	0.04 (0.02)	0.78 (0.05)	9.08 (−1.41)	3.10 (−0.99)	0.00 (−0.05)	29.15 (−9.19)	0.00 (0.00)
2	SFUD	36.32 (−0.09)	0.01 (0.00)	0.72 (−0.04)	15.67 (−1.39)	1.14 (−1.06)	0.00 (0.00)	39.12 (1.79)	0.00 (0.00)
	IMPT	36.41 (−0.01)	0.01 (0.00)	0.74 (−0.02)	9.87 (−1.27)	0.20 (−0.85)	0.00 (0.00)	34.74 (2.50)	0.00 (0.00)
3	SFUD	36.61 (0.17)	0.06 (0.00)	0.55 (−0.04)	34.65 (0.44)	19.26 (0.63)	2.4 (−0.11)	17.22 (−1.95)	1.04 (−0.17)
	IMPT	36.56 (0.03)	0.02 (0.00)	0.71 (0.02)	23.94 (0.65)	9.69 (−0.03)	0.15 (−0.01)	17.81 (−3.08)	0.13 (0.06)
4	SFUD	35.36 (−1.15)	0.03 (0.02)	0.70 (−0.03)	21.98 (0.46)	7.91 (0.67)	0.19 (0.00)	17.31 (−6.98)	0.00 (0.00)
	IMPT	36.12 (−0.41)	0.02 (0.01)	0.77 (0.04)	17.20 (0.82)	6.77 (1.07)	0.20 (0.07)	16.37 (−6.25)	0.00 (−0.01)
5	SFUD	36.20 (−0.32)	0.02 (0.01)	0.59 (−0.01)	9.64 (−3.65)	0.96 (−0.88)	0.00 (0.00)	25.89 (1.64)	0.00 (0.00)
	IMPT	36.31 (−0.19)	0.01 (0.00)	0.59 (0.00)	7.12 (−2.65)	0.77 (−0.66)	0.00 (0.00)	24.15 (2.03)	0.00 (−0.02)
6	SFUD	36.19 (−0.42)	0.02 (0.01)	0.69 (0.05)	13.44 (−7.42)	4.46 (−4.49)	0.00 (−0.54)	11.73 (0.75)	0.00 (0.00)
	IMPT	36.40 (−0.18)	0.01 (0.00)	0.69 (0.03)	11.37 (−6.98)	3.81 (−4.22)	0.00 (−0.48)	10.42 (0.80)	0.00 (−0.03)
7	SFUD	35.91 (−0.56)	0.02 (0.01)	0.73 (0.02)	10.28 (−2.97)	0.62 (−0.85)	0.00 (0.00)	32.28 (9.65)	0.00 (0.00)
	IMPT	36.14 (−0.34)	0.02 (0.01)	0.72 (0.01)	6.29 (−2.86)	0.16 (−0.46)	0.00 (0.00)	31.89 (9.84)	0.00 (0.00)
8	SFUD	35.93 (−0.67)	0.02 (0.01)	0.69 (0.03)	12.89 (−6.88)	0.98 (−2.69)	0.00 (−0.05)	19.16 (6.59)	0.00 (0.00)
	IMPT	35.82 (−0.71)	0.02 (0.01)	0.67 (−0.01)	6.47 (−6.44)	0.27 (−1.78)	0.00 (−0.03)	17.78 (6.28)	0.00 (0.00)
9	SFUD	35.49 (−0.94)	0.03 (0.02)	0.59 (−0.07)	8.47 (−6.12)	0.83 (−2.77)	0.00 (−0.01)	14.28 (3.40)	0.00 (0.00)
	IMPT	35.26 (−1.25)	0.03 (0.02)	0.60 (−0.07)	5.83 (−5.77)	0.34 (−2.34)	0.00 (0.00)	12.67 (3.39)	0.00 (−0.01)
10	SFUD	36.32 (−0.26)	0.01 (0.00)	0.61 (−0.03)	14.41 (0.59)	2.40 (0.80)	0.00 (0.00)	32.26 (11.54)	0.00 (0.00)
	IMPT	35.97 (−0.48)	0.02 (0.01)	0.66 (−0.06)	6.05 (1.34)	1.17 (0.84)	0.00 (0.00)	22.75 (9.18)	0.00 (0.00)

*Abbreviations:* CI, conformity index; CTV, clinical target volume; HI, homogeneity index; IMPT, intensity-modulated proton therapy; pp, percentage point; SFUD, single-field uniform dose.

*Note:* Data are presented as absolute accumulated dose metrics together with the corresponding differences (Δ_accum_). For dose metrics evaluated as percentages, absolute values are expressed as %, and Δ_accum_ values are expressed in percentage points (pp).

In terms of Metric_accum_, the dose constraint of rectum V36 was not achieved in 1 patient with the SFUD plan, whereas it was achieved in all patients with the IMPT plan.

The paired differences between the SFUD and IMPT plans for these Metric_accum_ are shown in Supplementary Figure S1. Among the evaluated metrics, the largest difference was observed in rectum V18.1, with a median difference of −4.39 pp.

## Discussion

In this study, we comprehensively evaluated the robustness of bs-PTV-based SFUD and robustly optimized IMPT plans for prostate cancer treated with a UHF schedule. To ensure the clinical realism of our dataset, we quantified the inter-fractional displacements of the gold marker (Supplementary Table S1). Notably, the largest shift in the AP direction and minimal shift in the LR direction were consistent with a prior report.^38^ Under setup and range uncertainties, the median difference in Δ_worst_ for CTV D99 between SFUD and IMPT plans was 0.11 GyE, corresponding to approximately 0.3% of the prescribed dose (36.25 GyE). Overall, target coverage was achieved within commonly accepted robustness criteria in both approaches.^39^ For OAR, statistically significant differences in Δ_worst_ between the SFUD and IMPT plans were observed in some dose metrics. Regarding the number of cases with unachieved dose constraints, SFUD plans recorded 2 cases each for rectum V36, bladder V18.1, and femoral head V14.5 and 1 case for rectum V29, whereas IMPT plans recorded 2 cases each for rectum V36 and bladder V18.1 and only 1 case for femoral head V14.5. Overall, the IMPT plans demonstrated a slightly lower number of unachieved dose constraints than SFUD plans, suggesting a slight clinical advantage in OAR sparing.

Notably, the absolute values of Δ_accum_ for both the rectum and bladder tended to be smaller than those of Δ_worst_. This discrepancy can be explained by the methodological differences between the 2 robust evaluation approaches. In the worst-case analysis, the maximum dose to OAR or minimum dose to target was selected from 20 setup and range uncertainty scenarios, resulting in larger deviations from the nominal dose. In contrast, the accumulated dose analysis was based on the summation of daily doses recalculated on 5 vCT images, in which day-to-day anatomical variations were likely averaged out.^40^ As a result, the impact of random anatomical changes on dose deviations was mitigated, leading to smaller Δ_accum_ values compared with Δ_worst_.

Interestingly, while SFUD plans exhibited slightly superior robustness for the CTV, IMPT plans showed slightly improved robustness compared with SFUD plans for several OAR dose metrics, which contrasts with the conventional perception that SFUD plans are inherently more robust for both target coverage and OAR sparing. This difference may be explained by the differing uncertainty management strategies. SFUD plans based on the bs-PTV may be insufficient for OAR near the target, particularly under setup and range uncertainties.^41^ In contrast, IMPT plans utilizing robust optimization consider inter-beam interactions and minimize target dose deviations under worst-case scenarios.^42^ Although OAR constraints were not explicitly incorporated into the robust optimization objectives in our study, applying robust optimization to the CTV may have contributed to the slightly improved robustness of OAR doses. Had robust optimization been applied to the SFUD plans, the dosimetric differences between the 2 techniques may have been reduced. However, it is equally possible that the observed differences simply reflect the overall dosimetric characteristics of the generated plans. Therefore, the advantages of IMPT observed in the present study reflect the combined benefit of the IMPT technique and the robust optimization strategy, rather than the inherent superiority of IMPT alone.

In the nominal, worst-case, and accumulated dose comparisons, IMPT plans consistently achieved lower rectum V18.1, rectum V29, and bladder V18.1 compared with SFUD plans. When target robustness is preserved, these reductions in OAR dose may represent a clinically meaningful advantage, particularly in the UHF schedule where each fraction delivers a high dose. Although previous studies have suggested that late gastrointestinal and genitourinary toxicities are more strongly associated with high-dose metrics of OAR,^43^, ^44^ reducing low- and intermediate-dose volumes may still contribute to overall toxicity reduction. Therefore, IMPT may offer a dosimetric benefit without compromising robustness in the UHF schedule.

While prostate proton therapy generally utilizes 2 opposed lateral fields, we employed a 4-field configuration (75°, 100°, 260°, and 285°) in this study to minimize the dosimetric impact of gold markers.^31^ This geometrical arrangement may influence overall plan robustness. In IMPT, increasing the number of fields provides greater degrees of freedom during optimization and reduces single-beam contributions. This, in turn, enables geometric compensation for range or setup errors along a beam path through the remaining fields. Thus, the observed robustness advantage of IMPT might potentially be generalizable across other configurations. Furthermore, this advantage might become even more pronounced with a conventional 2-field configuration. In such a configuration, the dosimetric impact of uncertainties along a single beam path is amplified, rendering bs-PTV-based SFUD more sensitive to uncertainties. In contrast, robustly optimized IMPT effectively maintains plan robustness through simultaneous multi-field optimization. Consequently, the robustness superiority of IMPT over SFUD might be further enhanced in a standard 2-field configuration. However, the robustness results may depend on the beam arrangement, and as this study did not quantitatively evaluate other configurations, further studies using different clinically relevant beam configurations are needed to confirm this hypothesis.

The pencil beam algorithm used in this study has inherent limitations in handling tissue heterogeneities at pelvic bone interfaces compared to Monte Carlo (MC) calculations. Nevertheless, its accuracy remains relatively high for prostate cases, with gamma pass rates of 99% or higher under the criteria of 3% and 3 mm.^45^ Furthermore, following our clinical protocol, gold fiducial markers were overridden with surrounding soft tissue densities, indicating that dose shadowing effects^46^ were not explicitly modeled in the dose calculations. As the primary objective of the present study was a relative comparison between SFUD and IMPT plans evaluated under the same algorithm, the validity of the observed trends and our main conclusions remain well maintained. Nevertheless, further validation using MC simulations would be valuable for future investigations.

This study has several limitations that should be acknowledged. First, the sample size was limited to ten patients, which reduces statistical power and generalizability. However, consistent trends were observed across patients. Second, the CBCT images used for dose recalculation did not cover the entire patient anatomy, and potential beam path changes due to body contour variations were not fully considered. However, because the UHF schedule completes treatment within approximately 1 to 2 weeks, substantial body shape changes are unlikely, and the resulting impact on the accumulated dose is expected to be limited. Nevertheless, as regions outside the wCBCT FOV were replaced with static pCT data, daily variations in the pelvic bone, femoral head, acetabular region, body contour, and soft-tissue composition along the beam path were not accounted for. Given that proton range and distal fall-off are highly sensitive to these variations, our CBCT-based dose accumulation may still underestimate the actual anatomical range uncertainties. Furthermore, these wCBCT images were obtained during a MHF schedule of 21 fractions, which may not adequately represent the anatomical variations occurring within a 1 to 2 week UHF schedule. While evaluating anatomical changes over a longer duration may capture a wider range of variations, such changes are not necessarily monotonic over treatment time. Nevertheless, the finding that the SFUD and IMPT plans maintained high robustness under these extended variations suggests that these approaches may be dosimetrically acceptable for an actual UHF schedule. Third, the vCT images used for dose recalculation represent only a single snapshot per fraction, and intra-fractional motion during irradiation was not accounted for. Tang et al investigated intra-fractional motion using Calypso tracking in 30 patients and reported no significant difference in dosimetric impact between SFO and MFO plans with average reductions in CTV D99 of less than 2% for both approaches,^47^ suggesting that the effect of intra-fractional motion is likely minimal in the present study. Finally, owing to the limited soft-tissue contrast of CBCT images, accurately identifying and contouring the urethra was extremely challenging. Consequently, we were unable to reliably calculate the urethral accumulated dose under anatomical changes using virtual CTs generated from CBCT images. Future studies are required to develop reliable methods to delineate the urethra on CBCT images. Nevertheless, our findings suggest that IMPT plans can achieve acceptable target robustness while providing slightly improved OAR sparing compared with SFUD plans. Therefore, IMPT plans with a robust optimization strategy may represent a potentially advantageous approach.

## Conclusion

Both bs-PTV-based SFUD and robustly optimized IMPT maintained adequate target coverage under uncertainties in prostate cancer treated with a UHF schedule, although IMPT achieved lower nominal OAR doses and slightly improved robustness. However, this advantage likely reflects the combined effects of differing planning techniques and uncertainty management strategies rather than a dosimetric superiority of IMPT. While robustly optimized IMPT offers a modest dosimetric benefit, future investigations are required to clarify the essential dosimetric differences between SFUD and IMPT.

## Ethics

The study design was approved by our institutional review board (approval number: 022–0076).

### Funding

This work was supported by JSPS KAKENHI Grant Number JP24K18816 and Grants-in-Aid for Regional R&D Proposal-Based Program from the Northern Advancement Center for Science & Technology of Hokkaido, Japan.

## CRediT authorship contribution statement

Hiroshi Tamura: Data curation, Image dataset preprocessing and organization, Methodology - treatment planning development, Formal analysis, Writing - original draft. Takaaki Yoshimura: Writing - original draft. Keiji Nakazato: Image dataset preprocessing and organization. Hiroto Yoshimoto: Image dataset preprocessing and organization. Seishin Takao: Methodology - treatment planning development. Taeko Matsuura: Methodology - treatment planning development. Takashi Mori: Methodology - treatment planning development, Writing - original draft. Kentaro Nishioka: Writing - original draft. Keiji Kobashi: Writing - original draft. Takayuki Hashimoto: Writing - original draft. Hidefumi Aoyama: Supervision. All authors read and approved the final manuscript.

### Data availability

The data supporting the findings of this study are not publicly available due to ethical restrictions but may be shared by the corresponding author upon reasonable request.

## Declaration of Conflicts of Interest

The authors declare that they have no known competing financial interests or personal relationships that could have appeared to influence the work reported in this paper.

## Footnotes

Appendix A Supplementary data associated with this article can be found in the online version at doi:10.1016/j.ijpt.2026.101951.
