# Supplementary material for: Clinical Comparison of Beam-Specific Planning Target Volume Single-Field Uniform Dose and Robust Intensity-Modulated Proton Therapy in Ultra-Hypofractionation for Prostate Cancer
Source: Int J Part Ther. 2026 Jul 15;21:101951. doi: 10.1016/j.ijpt.2026.101951 (PMC13449344; doi:10.1016/j.ijpt.2026.101951)
Supplement: Supplementary file 1 [file mmc1.docx]

Supplementary Figure S1. Boxplots of the paired dosimetric differences (IMPT minus SFUD) for CTV and OAR metrics. The evaluations were performed across nominal (blue), worst-case (red), and accumulated (light green) doses. Positive values indicate that the IMPT plan resulted in a higher dose or larger volume compared to the SFUD plan.

Supplementary Table S1. Median and range (minimum–maximum) of positional displacements of the gold marker relative to the pelvic bony anatomy in the LR, SI, and AP directions over five fractions for each patient.

| Patient | LR [mm] | | |  | SI [mm] | | |  | AP [mm] | | |
| --- | --- | --- | --- | --- | --- | --- | --- | --- | --- | --- | --- |
|  | **Median** | **Min** | **Max** |  | **Median** | **Min** | **Max** |  | **Median** | **Min** | **Max** |
| 1 | 1.5 | -0.4 | 2.4 |  | -1.0 | -2.0 | 0.0 |  | 2.1 | 0.8 | 4.7 |
| 2 | 0.4 | 0.2 | 1.2 |  | 0.1 | -0.1 | 0.1 |  | -0.7 | -1.1 | 1.0 |
| 3 | 0.4 | -1.0 | 4.3 |  | 1.0 | -1.0 | 2.0 |  | -0.4 | -2.0 | 0.5 |
| 4 | 0.5 | 0.2 | 0.9 |  | 1.0 | -3.0 | 4.0 |  | 0.2 | -1.1 | 4.5 |
| 5 | -0.3 | -0.9 | 3.2 |  | 1.0 | -1.0 | 2.0 |  | -0.7 | -1.6 | 0.0 |
| 6 | 0.9 | 0.2 | 1.3 |  | 1.0 | -1.0 | 3.0 |  | 0.1 | -0.8 | 2.1 |
| 7 | 0.7 | 0.4 | 1.0 |  | -2.0 | -5.0 | -0.7 |  | 3.8 | 2.1 | 5.6 |
| 8 | 0.5 | 0.2 | 0.9 |  | -2.0 | -4.0 | -1.0 |  | 4.5 | 2.5 | 9.4 |
| 9 | 0.0 | -0.3 | 0.8 |  | -1.0 | -2.0 | 1.0 |  | 3.9 | 1.3 | 6.1 |
| 10 | 0.0 | 0.0 | 0.3 |  | -1.0 | -2.0 | 0.0 |  | 0.0 | -2.3 | 2.1 |

**Abbreviations:** AP, anterior-posterior; LR, left-right; SI, superior-inferior.

**Note**: Positive values indicate shifts in the left, superior, and posterior directions, respectively.
